# Behavior of Hypochlorite and Chloramine-T

**Published:** 1918-12

**Authors:** 


**Behavior of Hypochlorite and Chloramine-T Solutions in Contact with Necrotic and Normal Tissues** *in vivo*. By J. Harold AUSTIN, M.D., and Herbert D. TAYLOR, M.D. *Journal of Experimental Medicine*. May 2, 1918.

Chlorine content, and consequently the disinfecting power of hypochlorite of soda and chloramine-T solutions, lessens on contact with the surface of wounds. This has some importance for the efficacity of the dressings performed with these antiseptics, and the necessity of their frequent renewal.

Experimental measures of the fall in chlorine concentration were effected in the following way.

Gangrenous wounds were developed on the left ears of 3 rabbits by a 20 minute exposure to the rays emitted by a Coolidge tube. After some weeks, when the wound had attained a certain size, following treatment was given : the gangrenous and normal ears were each placed separately for 20 minutes in beakers containing the solutions to be tested.

These solutions were : for rabbit N° 1, Dakin's hypochlorite solution; for rabbit N° 2, a sodium carbonate and sodium bicarbonate solution of comparable alkalinity with Dakin's solution; for rabbit N° 3, chloramine-T solution.

The following table gives the results of the experiments. Figures represent the number of cc. of 0.1 N sodium thiosulfate solution required to reduce the chlorine in 10 cc. of the antiseptic solution.

| SOLUTION | IN CONTACT WITH | Before. | Immediately after. | 2 hrs. after. | 17 hrs. after. |
|---|---|---|---|---|---|
| N° 1 | Normal ear | 13.00 | 12.35 | 12.15 | 11.50 |
| | Gangrenous ear | 13.00 | 11.55 | 10.30 | 8.65 |
| | Control (no tissue) | 13.00 | 13.00 | 13.05 | 12.60 |
| N° 2 | Normal ear | | | | |
| | Gangrenous ear | | | | |
| N° 3 | Normal ear | 12.75 | 12.75 | | 12.75 |
| | Gangrenous ear | 12.75 | 12.75 | | 12.35 |
| | Control | 12.75 | 12.75 | | 12.75 |

*Dakin's Solution*. A faint spontaneous fall in chlorine is marked by the control test. The stronger fall in chlorine noted in the solutions in contact with normal tissues is due to the erosive effect of that solution on hairs and epithelial cells. The very strong fall in chlorine concentration appearing in the solution in contact with necrotic tissues is due to the fact that the solution has a strong erosive action on the wound; this being proved by the gradual attack of cells, flecks of pus... by the available chlorine. This combines with the products of the tissues detached from the wound, dissolves all free particles, and produces with them stable combinations escaping the reduction by thiosulfate.

*Chloramine-T Solution* has a greater stability and no erosive effects either on sane or on necrotic tissues.

The repeated action of these solutions was also tested by the authors. The ears of each rabbit were suspended in solutions of the same types and concentrations as those used formerly for 7 consecutive days and during 20 minutes every day. After this the gangrenous wound treated with Dakin's solution had improved much more than the other gangrenous ears, proving the greater cleansing power of that solution.

The normal ears of the chloramine-T and alkaline solutions had undergone no changes.

The normal ear tested with Dakin's solution was intensely inflamed and showed superficial alterations in some places. The authors conclude, therefore, that the irritating effects must be due to available chlorine.

